# Handling Missing Data in the Short Form–12 Health Survey (SF-12): Concordance of Real Patient Data and Data Estimated by Missing Data Imputation Procedures

**DOI:** 10.1177/1073191120952886

**Published:** 2020-08-30

**Authors:** Markus A. Wirtz, Nicole Röttele, Matthias Morfeld, Elmar Brähler, Heide Glaesmer

**Affiliations:** 1 University of Education Freiburg, Freiburg im Breisgau, Germany; 2 Magdeburg-Stendal University of Applied Sciences, Stendal, Sachsen-Anhalt, Germany; 3 University of Leipzig, Leipzig, Germany; 4 University Medical Center, Mainz, Germany

**Keywords:** missing data, imputation methods, expectation-maximization-procedure, Short Form–12 health survey, health-related quality of life

## Abstract

If information on single items in the Short Form–12 health survey (SF-12) is missing, the analysis of only complete cases causes a loss of statistical power and, in case of nonrandom missing data (MD), systematic bias. This study aimed at evaluating the concordance of real patient data and data estimated by different MD imputation procedures in the items of the SF-12 assessment. For this ends, MD were examined in a sample of 1,137 orthopedic patients. Additionally, MD were simulated (a) in the subsample of orthopedic patients exhibiting no MD (*n* = 810; 71%) as well as (b) in a sample of 6,970 respondents representing the German general population (95.8% participants with complete data) using logistic regression modelling. Simulated MD were replaced by mean values as well as regression-, expectation-maximization- (EM-), and multiple imputation estimates. Higher age and lower education were associated with enhanced probabilities of MD. In terms of accuracy in both data sets, the EM-procedure (ICC_2,1_ = .33-.72) outperformed alternative estimation approaches substantially (e.g., regression imputation: ICC_2,1_ = .18-.48). The EM-algorithm can be recommended to estimate MD in the items of the SF-12, because it reproduces the actual patient data most accurately.

## Background

Measures of patient reported health-related quality of life (HRQoL) are key indicators of patient’s health condition. According to World Health Organization (WHO) standards, HRQoL must be considered as a third outcome parameter, in addition to mortality and morbidity, to ensure adequate and comprehensive measurement of patient’s health (WHO, 1995; Fayers & Machin, 2007). Assessment scales measuring patient reported HRQoL, like the Short Form–12 (Ware et al., 2001; Wirtz et al., 2018a), consist of several items. If respondents refuse to provide information on at least one of the scale items, the validity of the findings may be compromised by these missing data (MD) systematically. Generally, MD may lead to reduced test power. Furthermore, biasing effects have to be regarded if MD result from systematic, nonrandom causes (Allison, 2001; Schafer & Graham, 2002).

Missing data are defined as unexpected omitted information if complete collection designs are used (Graham, 2009). Although it can be assumed that the according information could have been reported people skipped or refused to mark a suitable response option. But statistical analyses and inferences assume data to be complete: The sampling procedure should determine which people constitute the analysis sample exclusively. But, if study participants are deleted from the study sample in case of MD (listwise deletion [LD] procedure) participant’s responses influence the composition of the study sample. Hence, the representativeness of the sample may be affected (Allison, 2001; Enders, 2010; Little & Rubin, 2002; Schafer & Graham, 2002; Wirtz, 2004). Missing data theory points out, that biasing effects arise if variables exist which are associated to the unexpected absence of information. Only if no systematic information (e.g., health state in HRQoL assessments) determines the occurrence of MD, the MD process can be characterized as missing-completely-at-random (MCAR; Graham, 2009). If data information is missing systematically and the probability of MD can be appropriately modelled by other variables in the data set, the MD process is denoted as missing-at-random (MAR). Nonrandom missing prevails if data are missing systematically and the probability of missing responses cannot be modelled by other variables in the data set. If the assumption of MCAR is inappropriate classical approaches like pairwise deletion or LD cause statistical biases. However, modern imputation techniques have been developed which allow for correcting such biases in case of MAR (Enders, 2010; Graham, 2009; Schafer & Graham, 2002). Taking into account systematic information associated with the occurrence of MD, regression, maximum-likelihood, or (Bayesian) multiple imputation (MI) procedures determine most plausible or likely estimates. Thus, a loss of statistical power as well as biases due to MD can be avoided in a reasonable and sound manner. Note, that for multi-item scales several items reflect the same underlying latent construct. Hence, items are correlated substantially. If single items were not answered and the absence is related to the underlying trait value, the refusal to answer can be systematically predicted from the remaining scale items. Hence, the assumption of MAR is highly plausible for HRQoL scales (Ayilara et al., 2019; Enders, 2010; Fielding et al., 2016).

Handling of MD in the assessment of HRQoL according to modern standards has mainly been fostered by empirical analysis and simulation studies on data collected in randomized control trials (RCT; Schulz et al., 2010). If missing information is not correctly taken into account in the analysis, this has a biasing effect on the measurable effect of health interventions (Bell et al., 2014; Fielding et al., 2008; Fielding et al., 2009; Fielding et al., 2016; Wood et al., 2004). In particular, thorough MD diagnostics, identification of underlying MD processes, imputation procedures, and sensitivity analyses are considered the gold standard when dealing with MD (Carpenter & Kenward, 2008; Thabane et al., 2013). Nevertheless, there are considerable deficits in the practical application. For example, Fielding et al. (2016) found, that in only 23% of the studies MD processes are discussed. Nearly half of the studies applied LD despite its conceptual inadequacy. For several HRQoL scales, empirical evidence of the superiority of maximum likelihood-based and Bayesian procedures compared with classical standard procedures, like mean value replacement (MVR), pairwise deletion and LD, could be confirmed (e.g., EuroQoL; Simons et al., 2015; WHO Quality of Life Abbreviated Questionnaire, Lin, 2006; Western Ontario and Mc Master Osteoarthritis Index, Ghomrawi et al., 2011). For the Short Form–36 health survey (SF-36) and its Short Form–12 health survey (SF-12) mainly sensitivity analyses were conducted to determine unbiased estimates in randomized control trials and to determine estimation accuracy (Fielding et al., 2016; Rombach et al., 2016). Accordingly, full information maximum likelihood estimation, Bayesian estimates and MI are recommended imputation procedures for both instruments (Ayilara et al., 2019; Biering et al., 2015; Gomes et al., 2016, Halme & Tannenbaum, 2018, Peyre et al., 2011). Furthermore, using these techniques is suggested because data have to be assumed to be MAR (Fielding et al., 2009; Liu et al., 2005; Morfeld et al., 2003; Perneger & Burnand, 2005). In clinical settings elderly people and people with lower educational background exhibit enhanced probabilities of missing responses. In nonclinical settings for elderly people and for women with pronounced health impairments, an enhanced risk of MD was reported (Perneger & Burnand, 2005).

But in practice, largely inconsistent recommendations are given for handling MD in the SF-36 and SF-12. Perneger and Burnand (2005) as well Hopman et al. (2011) recommend MVR, despite its conceptual inadequacy (see also Fairclough, 2002; Morris & Coyle, 1994). Complete case analysis is recommended in the original manuals of the instrument (Morfeld et al., 2011; Ware et al., 2001). But this turns out to be unjustifiable, especially, because up to 50% of the respondents have MD on at least 1 of the 12 items (Ware et al., 2001). Clinical studies report substantial rates of 14% to 31% of MD for the SF-12.

Hence, this study strives to empirically analyze the properties of generally recommended imputation techniques for the SF-12 data on item-level in a clinical as well as in a representative normative sample of German adults. The capability of the imputation procedures to adequately reproduce real patient data on the individual items of the SF-12 is examined. Since the patient data are actually known, the validity and accuracy of the imputed values can be determined item-specifically with regard to their concordance with the actual values. To enable this analysis strategy, in a first step a MD analysis will be conducted in order to identify patterns of MD as well as data information associated with missing information (Allison, 2001; Graham, 2009). Subsequently, for participants with complete data in both data sets the occurrence of MD will be simulated. Third, the validity and accuracy of mean-, regression-, expectation-maximization (EM-) as well as (Bayesian) MI will be evaluated in terms of concordance of real patient data and imputed data for both data sets independently. This approach corresponds to that adopted by Perneger and Burnand (2005) as well as Hopman et al. (2011), who analyzed the appropriateness of MVR imputation for the SF-12 items. We expect that the model-based imputation techniques EM- as well as Bayesian-imputation, which are recommended if the MD process is MAR, outperform classical imputation approaches as mean- or regression-imputation. These findings should help to improve the existing recommendations for handling MD in SF-12 in accordance with the MD theory and clinical and epidemiological data.

## Method

### Data Collection and Study Population

#### Clinical Sample of Orthopedic Rehabilitation Patients

Data were collected in six rehabilitation institutions in a study founded by the leading associations of statutory health and pension insurance in Germany (Bürger et al., 2002). A total of 1,176 patients suffering from orthopedic disease and taking part in a stationary or ambulatory treatment were included (excluded: rheumatic diseases). Because 39 patients (3%) answered no single item, the analysis sample was composed of 1,137 patients (45% women; age: 20 to 87 years; *M* = 49.74, *SD* = 11.34). 17% (*n* = 197) reported university entrance qualification, and 18% (*n* = 204) reported a secondary school certificate as highest educational school level.

#### Normative Sample of the German General Population

Data were collected within the German Health Survey 1997/1998 by the Robert Koch Institute (founded by the federal ministry of health) in 120 German communities (Radoschewski & Bellach, 1999). A random procedure based on information available by the residents’ registration office was applied. 7,124 people agreed to take part in the study (response rate: 61%), 6,790 people (98%) completed the SF-12 (51% women; age 17 to 79 years; *M* = 45.62, *SD* = 15.87). 37% (*n* = 2,565) reported university entrance qualification, and 20% (*n* = 1,420) reported a secondary school certificate as highest educational school level.

### Questionnaire Content

The German Health Survey 1997/1998 contained items on health-related behavior, life conditions and diseases. In the clinical sample sociodemographic characteristic, disease-related information, preceding treatments, characteristics of the occupational and social environment, characteristics of the rehabilitation treatment, the *Hannover scale of functional capacities* (Kohlmann & Raspe, 1996) and the *Symptom Checklist* (SCL-90; Franke, 1995) measuring subjectively perceived impairments caused by physical and mental symptoms were assessed.

In both samples the items of the SF-12 measuring HRQoL were answered by healthy as well as diseased persons older than 13 years (Wirtz et al., 2018b). Six items reflect physical aspects of HRQoL (PFI2, PFI4, ROLPH2, ROLPH3, PAIN2, GHP1), six items indicate mental health aspects (VITAL2, SOC2, ROLEM2, ROLEM3, MHI3, MHI4), respectively. Four items (ROLPH2, ROLPH3, ROLEM2, ROLEM3) exhibit a dichotomous answer format. The other items are answered on a 3- (PFI2, PFI4), 5- (GHP1, PAIN2) or 6- (MHI3, MHI4, VITAL2, SOC2) point Likert-type scale. All item answers range from 0 (*minimum health*) to 100 (*maximum health*; Ware et al., 2001; Wirtz et al., 2018a). Two scale scores *Physical Scale Score* and *Mental Health Score* can be calculated.

### Missing Data Diagnostics

For each SF-12 item a dummy variable indicating the absence of persons’ responses was defined: “0” = “missing”; “1” = “not missing.” To identify the underlying MD processes, Pearson correlations of the 12 dummy coded indicator variables and sociodemographic characteristics (age, gender, school graduation) as well as the *Hannover scale of functional capacities*, the SCL 90-R *Depression Scale* and the SF-12 *Physical* and *Mental scale scores* were determined.

### Simulation of Missing Values

Missing data were simulated in two steps. (a) *Identification of a logistic model predicting the occurrence of MD in the clinical data set*: Bivariate statistics and multivariate logistic regression models were used to specify an appropriate model predicting the occurrence of MD in the clinical sample. The logistically transformed probability of missing information is assumed to be a linear combination of a set of predictor variables (Gelman & Hill, 2007). (b) *Simulation of MD for complete data of the clinical and normative sample*: For each SF-12 item *i* and each participant *j*, the probability *p_ij_* of missing information was logistically determined based on the variables predicting missing information in the subsamples of participants with complete information in all SF-12 items. As participants with missing-associated predictor constellations were underrepresented in the subsample of participants answering all items completely, the relative frequencies of missing information were attenuated in the simulated data sets. The attenuation factor was defined as $p_{ij,adjust}=(p_{ij,logistic}*h_{i})/\;{\bar{p}}_{i},$ with *p_ij,logistic_* = logistically determined probability for item *i* and participant *j*, *p−*_i_ mean value of *p_ij,logistic_* on item i, and h_*i*_ = relative frequency of MD in item i in the original clinical data set. The correcting factor ensured similar frequency rates of missing in the simulation and the original samples. According to these empirically estimated probabilities, simulated indicator variables (SIV_*i*_) were calculated stochastically (“0” = “missing,” “1” = “not missing”). Hence, this calculation procedure allowed simulating the known MD processes for participants with complete data in an empirical-based and valid manner. For participants with SIV_*i*_ = 1 the original answer values were retained; for participants with SIV_*i*_ = 0 data were assigned as “missing.” The simulation was realized 10 times for each item to empirically evaluate the stability of the imputation procedures (Graham et al., 2007). Accordingly, 120 simulated data sets were generated for each of both data sets.

### Evaluation of Imputation Techniques

Seven imputation techniques were evaluated comparatively:

1. *Imputation by item mean value* (*IMV*): Missing data in item *i* were replaced by the mean value of the available answers on item *i*.
2. *Imputation by participants’ mean value* (*PMV*): Missing data of participant *j* were replaced by the mean value of the answers on the other scale items.
3. *Imputation by stochastic regression* (*SR_residual_, SR_t-student_*): Missing values were replaced by multiple regression estimates using the other SF-12 items as predictors. Residual error terms were added to account appropriately for stochastic components: *SR_residual_* adds an error drawn from the sample of participants with complete data. For *SR_t-student_* an error is drawn from a Student *t*-distribution (Enders, 2010; Little & Rubin, 2002).
4. *Imputation by EM-algorithm* (*EM*): Applying a maximum-likelihood-procedure, model parameters were determined within an iterative estimation process. Taking available empirical information into account the EM-algorithm ensures an optimal correspondence of model predictions and empirical information (Allison, 2001; Little & Rubin, 2002; Schafer & Graham, 2002).
5. *Multiple Imputation* (*MI_DA_, MI_FCS_*; Enders, 2010; Graham, 2009; Graham et al., 2007): The statistic model is similar to SR-imputation, but the underlying statistical distribution is estimated using a Bayesian approach. Two MI-approaches have been adopted: The data-augmentation-algorithm (MI_DA_) assumes a joint multivariate normal distribution of all analysis variables and imputes all information in a single imputation step (Shrive et al., 2006; van Buuren et al., 2006). The fully conditional specification algorithm (MI_FCS_) is a Markov chain Monte Carlo method utilizing a specific imputation model for each variable. Because in our analyses data are missing only for single items, in each simulation this approach is conceptually similar to MI_DA_.

Applying SR, EM, and MI, all items without MD (i.e., 11 of 12 variables) were used as predictors in the imputation model. Precision and validity of the imputation was evaluated by investigating the correspondence of the imputed data and the original participants’ answers. Following the approach of Perneger and Burnand (2005) intraclass correlation (ICC_2,1_; McGraw & Wong, 1996; Wirtz, 2017) was adopted as measure of correspondence, indicating absolute agreement of imputed and original values. To ensure reasonable average ICC-values, mean calculation was based on Fisher Z-transformed original ICC-values (Salkind, 2006). IBM SPSS AMOS 23.0 was utilized to estimate MD by *MI_DA_*. All other imputation procedures were realized using IBM SPSS 23.0.

## Results

### Missing Data Structure

Because the normative study sample ensured an uncommon elaborate data assessment (individually assisted data collection at home), the MD rates were far below typical rates (Liu et al., 2005; Morfeld et al., 2003; Perneger & Burnand, 2005): 6,943 (99.6%) of the 6,970 participants responded to at least 9 of the 12 items, and less than 1% of the data were missing for each item. In the clinical sample of orthopedic rehabilitation samples, substantial rates of MD prevailed (Table 1). However, 815 of the 1,137 study participants answered all 12 items of the SF-12 (MD rate: 28%). 161 participants (14 %) exhibited more than 3 missing values on the 12 items (missing rate > 30%).

**Table 1.** Frequency of Missing Values per Participant in the Sample of Orthopedic Patients (*n* = 1,137).

Number of missing data	Frequency		%_cum_
	*n*	%	
0	815	71.2	71.2
1	52	4.6	75.9
2	80	7.0	82.9
3	29	2.6	85.7
4	55	4.8	90.5
5	69	6.1	96.6
6	11	1.0	97.6
7	3	0.3	97.9
8	5	0.4	98.4
9	0	0.0	98.4
10	0	0.0	98.4
11	18	1.6	100.0

Table 2 depicts MD frequencies for each item. The following information on the distribution of MD refers only to the clinical sample. The Item “Did you have lots of energy?” (VITAL2) exhibited the highest frequency of MD (*N_missing_* = 202; 17.8%). The item on bodily pain (PAIN2) was answered most completely (*N_missing_* = 17; 1.5%). Generally, the items on *Physical health* were answered more completely than the items on *Mental health*. In the mental domain, only for the item on social functioning a high response rate was observed (*N_missing_* = 32; 2.8%).

**Table 2.** Frequency of Missing Values for Each SF-12 Item in the Sample of Orthopedic Patients (*n* = 1,137) and in the Normative Sample (*n* = 6,970).

			Clinical sample (*n* = 1,137)						Normative sample (*n* = 6,970)					
			Missing values		*M*	*SD*	*r*_it,c_^a^	α	Missing values		*M*	*SD*	*r*_it,c_^a^	α
Scale	Item content	Code	*n*	%					*n*	%				
Physical	Physical functioning: Moderate activities	PFI2	43	3.8	37.02	33.20	.55	.73	31	0.4	85.16	27.88	.64	.83
	Physical functioning: Climbing several flights of stairs	PFI4	93	8.2	50.28	35.45	.43		25	0.4	83.16	29.09	.58	
	Role physical: Accomplished less	ROLPH2	130	11.4	13.76	34.47	.48		47	0.7	79.59	40.31	.63	
	Role physical: Limited in the kind of work	ROLPH3	118	10.4	18.68	39.00	.50		61	0.9	85.04	35.67	.68	
	Bodily pain: Interfere with normal work	PAIN2	17	1.5	35.12	25.59	.59		58	0.8	50.89	17.97	.52	
	General health	GHP1	35	3.1	30.87	18.27	.34		4	0.1	78.62	26.24	.64	
Mental	Vitality: Lot of energy	VITAL2	202	17.8	39.53	24.16	.46	.77	49	0.7	57.60	24.28	.53	.79
	Social functioning: Interference of health aspects	SOC2	32	2.8	64.95	25.68	.46		64	0.9	84.27	22.28	.57	
	Role emotional: Accomplished less	ROLEM2	113	9.9	51.41	50.01	.67		39	0.6	86.73	33.92	.57	
	Role emotional: less carefully	ROLEM3	161	14.2	56.10	49.66	.65		56	0.8	89.62	30.50	.57	
	Mental health: Calm and peaceful	MHI3	127	11.2	54.06	23.73	.52		42	0.6	63.25	24.13	.50	
	Mental health: Downhearted an depressed	MHI4	118	10.4	67.72	22.09	.59		39	0.6	80.74	20.35	.58	

*Note*. PFI = physical functioning; ROLPH = role physical; PAIN = bodily pain; GHP = general health; VITAL = vitality; SOC = social functioning; ROLEM = role emotional; MHI = mental health.

a Corrected item-total-correlation.

Overall, 112 different MD patterns were identified. Except for single missing, no MD pattern appeared in more than six cases (1.8%). Because no MD profiles characterized respondents’ answering behavior predominantly, the following analyses focused on single item missing information.

Table 3 shows the correlation of missing indicator variables (“0” = “missing”; “1” = “not missing”) for each item with potentially relevant missing covariates. Despite item GHP1 (“General Health”), for all items missing information is associated with increasing age. Generally, a lower educational level corresponds to a higher risk of omitted information. For each health indicator a maximum of 6 missing indicator variables was significantly associated with a lower health state. To control for multivariate dependencies between gender, age, educational level, and health indicators, a multivariate logistic regression approach was adopted: For any item only age and educational level proved to be significantly associated with missing information (Table 4).

**Table 3.** Bivariate Correlations of Missing Information (Indicator Variables) and Sociodemographic Characteristic and Health State.

	Missing information: Indicator variables^a^											
	Physical scale						Mental scale					
	PFI2	PFI4	ROLPH2	ROLPH3	PAIN2	GHP1	VITAL2	SOC2	ROLEM2	ROLEM3	MHI3	MHI4
Gender	−.03	.02	−.01	.05	−.01	−.01	.04	−.02	**.06**	.02	**.12**	**−.09**
Age	**−.07**	**−.10**	**−.14**	**−.17**	**−.11**	−.05	**−.20**	**−.07**	**−.13**	**−.17**	**−.13**	**−.15**
University entrance qualification	.05	**.07**	**.09**	**.06**	.02	**.07**	**.10**	.05	**.10**	**.09**	**.08**	.04
Secondary school certificate	**.07**	.06	**.08**	.03	−.02	−.01	**.07**	.01	.03	**.06**	.02	**.09**
FFbH-R: Functional capacity	.01	−.02	**.11**	−.01	.04	−.01	**.13**	.02	**.08**	.03	**.12**	−.04
SCL-90-R: Depression scale	−.02	.05	.01	.02	−.01	.04	−.03	.05	−.03	.02	−**.12**	**.13**
SF-12: Physical scale	.03^b^	.01^b^	**.16** ^b^	.01^b^	.05^b^	−.04^b^	**.13**	.00	**.06**	**.06**	**.12**	−.04
SF-12: Mental scale	.05	.03	**.11**	−.01	.01	.01	**.18** ^b^	−.06^b^	**.13** ^b^	**.10** ^b^	**.21** ^b^	**−.13** ^b^

*Note. n =* 1,031 to 1,137. PFI = physical functioning; ROLPH = role physical; PAIN = bodily pain; GHP = general health; VITAL = vitality; SOC = social functioning; ROLEM = role emotional; MHI = mental health; FFbH-R = Hannover scale of functional capacities; SCL = symptom checklist; SF-12 = Short Form–12 health survey.

a 0 = missing; 1 = not missing. ^b^Scale values determined by the remaining 5 scale items.

*p* < .05 (values mentioned in bold face).

**Table 4.** Logistic Regression Weights *b* and Odds Ratios (*OR*) of Age and Dummy Coded Educational Level Predicting Missing Information in the SF-12 Items.

	Indicator variables: Missing information^a^																	
	PFI2_m			PFI4_m			ROLPH2_m			ROLPH3_m			PAIN2_m			GHP1_m		
	*b*	*SE b*	*OR*	*b*	*SE b*	*OR*	*b*	*SE b*	*OR*	*b*	*SE b*	*OR*	*b*	*SE b*	*OR*	*b*	*SE b*	*OR*
Age	−**0.03**	0.02	0.97	−**0.03**	0.01	0.97	−**0.04**	0.01	0.97	−**0.05**	0.01	0.95	−**0.09**	0.02	0.92	−0.02	0.02	0.98
SSC	**1.62**	0.73	5.07	**0.71**	0.35	2.03	**0.93**	0.32	2.54	0.32	0.28	1.38	−0.32	0.6	0.73	−0.01	0.44	0.99
UEQ	1.11	0.61	3.03	**0.84**	0.38	2.31	**1.01**	0.34	2.75	0.55	0.32	1.74	0.15	0.78	1.16	1.89	1.03	6.63
Constant	**4.50**	0.84	90.2	**3.7**	0.57	40.47	**3.65**	0.5	38.5	**4.67**	0.54	106.2	**9.14**	1.45	93.25	**4.31**	0.88	74.31
Nagelkerke *R*^2^			**.05**			**.04**			**.07**			**.07**			**.10**			**.03**
	Indicator variables: Missing information																	
	VITAL2_m			SOC2_m			ROLEM2_m			ROLEM3_m			MHI3_m			MHI4_m		
	*b*	*SE b*	*OR*	*b*	*SE b*	*OR*	*b*	*SE b*	*OR*	*b*	*SE b*	*OR*	*b*	*SE b*	*OR*	*b*	*SE b*	*OR*
Age	−**0.05**	0.01	0.96	−**0.03**	0.02	0.97	−**0.04**	0.01	0.97	−**0.04**	0.01	0.96	−**0.04**	0.01	0.97	−**0.04**	0.01	0.96
SSC	**0.64**	0.24	1.9	0.23	0.5	1.26	0.32	0.28	1.38	**0.54**	0.26	1.72	0.23	0.26	1.26	**1.08**	0.36	2.95
UEQ	**0.77**	0.26	2.15	1.05	0.74	2.87	**1.16**	0.4	3.18	**0.73**	0.29	2.07	**0.77**	0.33	2.16	0.4	0.3	1.49
Constant	**3.73**	0.42	41.8	**5.16**	0.96	174	**3.85**	0.53	47.2	**3.92**	0.46	50.36	**3.74**	0.5	42.08	**4.18**	0.53	65.54
Nagelkerke *R*^2^			0.09			**.03**			**.05**			**.07**			**.04**			**.07**

*Note. n* = 1,129. SF-12 = Short Form–12 health survey; PFI = physical functioning; ROLPH = role physical; PAIN = bodily pain; GHP = general health; *SE* = standard error; SSC = secondary school certificate. UEQ = university entrance qualification.

a 0 = missing; 1 = not missing.

*p* < .05 (values mentioned in bold face).

### Simulation of Missing Data

Table 4 shows the parameters of the logistic regression models predicting missing information determined in the clinical data set. Based on age and educational level, the probability of missing was determined. After correcting for the lower age and higher educational level in the subsample of participants with complete data, participants’ data were deleted randomly according to the probability of missing information. For each item here, 10 data sets were determined for the clinical sample (*n* = 810; 71%) as well as for the normative sample. Hence, 240 data sets were simulated containing item-specific MD replacements. Accordingly, each simulated data set was composed of (a) the imputed values replacing the simulated missing responses and (b) the original data otherwise.

### Empirical Evaluation of the Correspondence of Respondents’ Real Data and Imputed Data

Table 5 (clinical data) and Table 6 (normative data) depict the results for imputation concordance by ICC_2,1_ as measures of absolute agreement of imputed and real data (Perneger & Burnand, 2005). For the clinical as well as the normative data, the *EM-algorithm* allowed for the best data estimation (*mean* ICC_2,1_ = .33-.72). Because *IMV* estimates the same value for all cases, no systematic concordance prevails (ICC_2,1_ = 0). *PMV* yielded poor correspondence (*mean* ICC_2,1_ = .09-.30; exception: PAIN2).

**Table 5.** Intraclass Correlations of Imputed and Empirical Data in the Clinical Sample.

	PFI2					PFI4					ROLPH2					ROLPH3				
Imputation procedure	*Min*	*Max*	*Mdn*	*M*	*SD*	*Min*	*Max*	*Mdn*	*M*	*SD*	*Min*	*Max*	*Mdn*	*M*	*SD*	*Min*	*Max*	*Mdn*	*M*	*SD*
IMV	.00	.00	.00	.00	.00	.00	.00	.00	.00	.00	.00	.00	.00	.00	.00	.00	.00	.00	.00	.00
PMV	.05	.45	.24	.22	.10	.09	.26	.15	.17	.06	.02	.14	.11	.09	.04	.06	.24	.14	.15	.05
Regression (*t*-Student)	.12	.36	.24	.25	.07	.01	.25	.19	.18	.07	−.04	.44	.21	.21	.13	.00	.41	.19	.21	.11
Regression (Residual)	.09	.62	.44	.40	.16	.13	.33	.24	.24	.06	.12	.44	.31	.31	.11	.07	.44	.29	.27	.10
EM-algorithm	.17	.73	**.58**	**.54**	.15	.24	.46	**.34**	**.33**	.07	.30	.55	**.49**	**.46**	.09	.29	.55	**.43**	**.44**	.07
MI DA-algorithm	−.16	.20	−.01	.01	.11	.04	.35	.08	.14	.11	.07	.24	.14	.14	.07	−.01	.27	.10	.12	.10
MI FCS-algorithm	−.06	.28	.14	.13	.09	.02	.35	.14	.16	.11	−.13	.27	.13	.11	.12	.10	.33	.20	.20	.07
	PAIN2					GHP1					VITAL2					SOC2				
Imputation procedure	*Min*	*Max*	*Mdn*	*M*	*SD*	*Min*	*Max*	*Mdn*	*M*	*SD*	*Min*	*Max*	*Mdn*	*M*	*SD*	*Min*	*Max*	*Mdn*	*M*	*SD*
IMV	.00	.00	.00	.00	.00	.00	.00	.00	.00	.00	.00	.00	.00	.00	.00	.00	.00	.00	.00	.00
PMV	−.11	.73	.49	.46	.22	.10	.43	.33	.32	.11	.16	.28	.18	.20	.04	−.18	.53	.27	.27	.18
Regression (*t*-Student)	.03	.84	.38	.42	.24	−.25	.55	.27	.25	.23	.19	.41	.30	.29	.08	−.12	.52	.30	.29	.08
Regression (Residual)	−.34	.64	.46	.29	.34	−.07	.41	.25	.22	.13	.30	.44	.35	.36	.04	−.06	.73	.32	.34	.25
EM-algorithm	.31	.81	**.65**	**.59**	.17	.26	.58	**.42**	**.42**	.10	.43	.56	**.52**	**.51**	.03	.08	.75	**.44**	**.44**	.20
MI DA-algorithm	.04	.35	.08	.14	.11	.02	.48	.14	.18	.13	.23	.34	.27	.27	.03	.04	.48	.25	.28	.15
MI FCS-algorithm	−.19	.51	.29	.24	.20	−.06	.57	.19	.20	.16	.22	.43	.31	.31	.06	−.04	.39	.21	.20	.15
	ROLEM2					ROLEM3					MHI3					MHI4				
Imputation procedure	*Min*	*Max*	*Mdn*	*M*	*SD*	*Min*	*Max*	*Mdn*	*M*	*SD*	*Min*	*Max*	*Mdn*	*M*	*SD*	*Min*	*Max*	*Mdn*	*M*	*SD*
IMV	.00	.00	.00	.00	.00	.00	.00	.00	.00	.00	.00	.00	.00	.00	.00	.00	.00	.00	.00	.00
PMV	.24	.33	.29	.29	.03	.25	.32	.29	.29	.03	.27	.47	.38	.38	.06	.12	.35	.21	.22	.07
Regression (*t*-Student)	.34	.66	.48	.48	.09	.30	.50	.44	.43	.07	.23	.41	.30	.32	.05	.30	.44	.36	.36	.04
Regression (Residual)	.42	.66	.57	.57	.08	.39	.68	.50	.54	.09	.31	.49	.40	.40	.06	.31	.59	.39	.41	.08
EM-algorithm	.66	.79	**.72**	**.72**	.04	.64	.72	**.70**	**.71**	.04	.52	.65	**.59**	**.59**	.04	.47	.64	**.59**	**.58**	.05
MI DA-algorithm	.07	.36	.22	.21	.10	.27	.45	.40	.39	.05	.24	.46	.35	.34	.06	.09	.34	.24	.24	.07
MI FCS-algorithm	.19	.51	.37	.36	.10	.10	.34	.25	.25	.07	.11	.38	.20	.21	.09	.21	.43	.31	.33	.07

*Note. n* = 7 to 169; maximum values are in bold face. PFI = physical functioning; ROLPH = role physical; IMV = imputation by item mean value; PMV = imputation by participants’ mean value; PAIN = bodily pain; GHP = general health; VITAL = vitality; SOC = social functioning; ROLEM = role emotional; MHI = mental health; EM = expectation maximization; MI = multiple imputation; DA = data augmentation; FCS = fully conditional specification.

**Table 6.** Intraclass Correlations of Imputed and Empirical Data in the Normative Sample.

Imputation procedure	PFI2					PFI4					ROLPH2					ROLPH3				
	*Min*	*Max*	*Mdn*	*M*	*SD*	*Min*	*Max*	*Mdn*	*M*	*SD*	*Min*	*Max*	*Mdn*	*M*	*SD*	*Min*	*Max*	*Mdn*	*M*	*SD*
IMV	.00	.00	.00	.00	.00	.00	.00	.00	.00	.00	.00	.00	.00	.00	.00	.00	.00	.00	.00	.00
PMV	.22	.38	.30	.30	.04	.26	.35	.31	.30	.03	.22	.29	.26	.26	.02	.28	.32	.30	.30	.01
Regression (*t*-Student)	.27	.42	.36	.38	.07	.29	.41	.37	.36	.04	.41	.51	.45	.45	.03	.39	.54	.47	.47	.03
Regression (Residual)	.37	.55	.44	.46	.05	.38	.50	.43	.44	.03	.50	.59	.55	.54	.03	.51	.58	.55	.55	.02
EM-algorithm	.55	.63	**.60**	**.59**	.02	.54	.64	**.59**	**.58**	.03	.69	.74	**.71**	**.71**	.02	.67	.71	**.69**	**.69**	.01
MI DA-algorithm	.27	.44	.36	.36	.05	.24	.33	.26	.27	.03	.42	.49	.46	.46	.02	.38	.45	.43	.42	.02
MI FCS-algorithm	.33	.49	.44	.43	.04	.32	.39	.35	.35	.03	.43	.48	.45	.45	.01	.53	.59	.56	.56	.02
Imputation procedure	PAIN2					GHP1					VITAL2					SOC2				
	*Min*	*Max*	*Mdn*	*M*	*SD*	*Min*	*Max*	*Mdn*	*M*	*SD*	*Min*	*Max*	*Mdn*	*M*	*SD*	*Min*	*Max*	*Mdn*	*M*	*SD*
IMV	.00	.00	.00	.00	.00	.00	.00	.00	.00	.00	.00	.00	.00	.00	.00	.00	.00	.00	.00	.00
PMV	.51	.66	.62	.60	.04	.20	.39	.29	.28	.05	.19	.23	.22	.22	.01	.04	.22	.14	.14	.05
Regression (*t*-Student)	.27	.50	.37	.39	.07	.19	.36	.28	.28	.06	.33	.40	.36	.36	.02	.31	.49	.38	.39	.05
Regression (Residual)	.36	.60	.51	.50	.08	.27	.42	.33	.34	.05	.38	.45	.42	.42	.02	.33	.51	.46	.44	.05
EM-algorithm	.56	.71	**.65**	**.65**	.04	.44	.60	**.52**	**.52**	.04	.56	.60	**.58**	**.58**	.01	.49	.64	**.60**	**.60**	.05
MI DA-algorithm	.34	.58	.48	.47	.06	.23	.44	.31	.31	.06	.31	.39	.35	.35	.03	.28	.43	.37	.37	.04
MI FCS-algorithm	.44	.55	.47	.48	.03	.23	.42	.34	.34	.06	.39	.47	.42	.42	.02	.28	.46	.34	.35	.06
Imputation procedure	ROLEM2					ROLEM3					MHI3					MHI4				
	*Min*	*Max*	*Mdn*	*M*	*SD*	*Min*	*Max*	*Mdn*	*M*	*SD*	*Min*	*Max*	*Mdn*	*M*	*SD*	*Min*	*Max*	*Mdn*	*M*	*SD*
IMV	.00	.00	.00	.00	.00	.00	.00	.00	.00	.00	.00	.00	.00	.00	.00	.00	.00	.00	.00	.00
PMV	.16	.20	.18	.18	.01	.17	.21	.19	.19	.01	.21	.31	.24	.24	.03	.12	.17	.16	.16	.02
Regression (*t*-Student)	.33	.46	.38	.39	.04	.32	.43	.37	.37	.03	.20	.33	.25	.26	.04	.25	.35	.29	.30	.03
Regression (Residual)	.43	.51	.47	.47	.02	.39	.50	.43	.44	.03	.26	.36	.32	.29	.03	.28	.40	.34	.35	.03
EM-algorithm	.63	.70	**.65**	**.65**	.02	.60	.66	**.62**	**.62**	.02	.47	.53	**.49**	**.49**	.02	.42	.56	**.53**	**.52**	.03
MI DA-algorithm	.21	.33	.25	.25	.04	.20	.27	.23	.23	.02	.17	.28	.21	.21	.03	.20	.28	.24	.24	.02
MI FCS-algorithm	.36	.42	.40	.39	.02	.29	.38	.34	.35	.03	.29	.37	.34	.34	.03	.20	.36	.31	.30	.04

*Note. n* = 92 to 1,202; maximum values are in bold face. PFI = physical functioning; ROLPH = role physical; IMV = imputation by item mean value; PMV = imputation by participants’ mean value; PAIN = bodily pain; GHP = general health; VITAL = vitality; SOC = social functioning; ROLEM = role emotional; MHI = mental health; EM = expectation maximization; MI = multiple imputation; DA = data augmentation; FCS = fully conditional specification.

In the normative sample *stochastic regression imputation* (residual; mean ICC_2,1_ = .29-.55) and *MI* (FCS-algorithm; mean ICC_2,1_ = .30-.56) allowed for the second best estimation. Overall, *stochastic regression imputation* (*t*-student; mean ICC_2,1_ = .26-.47) and *MI* using DA-algorithm yield considerable lower correspondence of imputed and real data. In the clinical data set only *stochastic regression imputation* (*t*-student; mean ICC_2,1_ = .18-.48) and *both MI techniques* (mean ICC_2,1;FCS_ = .13 - .39; mean ICC_2,1;FCS_ = .01-.30) performed considerably worse.

## Discussion

Different MD imputation approaches were applied for the items of the SF-12 assessment. The accuracy of the imputation procedures was evaluated in terms of concordance of patients’ actual responses and imputed data (Perneger & Burnand, 2005). Current scoring rules recommend eliding respondents’ data in case of any missing responses (LD; Morfeld et al., 2011; Ware et al., 2001) or using IMV or PMV (Hopman et al., 2011; Perneger & Burnand, 2005). LD causes a considerable loss of statistical power. In line with previous studies (Liu et al., 2005; Morfeld et al., 2003) our results revealed, that using LD leads to a more likely removal of elderly and lower educated people from the study sample. Thus, MD processes are not MCAR: applying LD modifies the sample structure and systematic analytical biases have to be expected (Allison, 2001; Enders, 2010; Graham 2009; Schafer & Graham, 2002). Multivariate logistic modelling suggests that the bivariate association of lower health state and MD can be assumed to be indirectly caused by confounding higher age and lower educational level of people suffering from higher health impairments.

To comparatively validate the conceptual and statistical superiority of modern imputation techniques in case of violations of MCAR (Allison, 2001; Graham, 2009) over LD as well as elementary imputation procedures (MVR [IMV, PMV], SR), an empirical simulation approach was adopted (Perneger & Burnand, 2005). We analyzed if imputation procedures master reproducing participants’ real data appropriately. To this end, a logistic approach was applied, simulating the identified MD processes in the clinical data set of orthopedic rehabilitation patients. Participants’ answers were deleted stochastically according to the logistically predicted probability of missing responses. To determine the variability of the imputation estimates for each item, 10 data sets were simulated in the clinical as well as in the normative sample. In terms of absolute agreement of participants real and imputed data, the EM algorithm (mean ICC_2,1_ = .33-.72) outperformed all other imputation procedures for all 12, SF-12 items consistently. The EM algorithm provides reasonable to good estimates of participants’ answers, if participants’ answers would have been refused due to the empirical determined and simulated MD process.

At the item level, it should be noted that the quality of imputation is related to the item total correlation of the items. Considering the clinical sample, the items ROLEM2 and ROLEM3 exhibit the highest item total correlations *r_it_* = .67 and .65 (Table 2) and the ICC shows the highest concordance of EM-imputed values and actual values with *mean* ICC = .72 and .71, respectively (Table 5). For the items VITAL2 and SOC2, both the item total correlation (*r_it_* = .46 in each case) and the imputation quality (*mean* ICC = .51, .44) are significantly lower. For the items of the *Physical health* scale, the discriminatory power also corresponds to the average ICCs: PFI2 and PAIN can be imputed most accurately (*mean* ICC = .58, .59) and have the highest item total correlation (*r_it_* = .55, 59). Fisher Z-transformed corrected item-scale correlations and the Fisher Z-transformed *mean* ICCs correlate very highly with a value of .89 (*p* < .01). The normative sample also shows a positive, however, not significant correlation (*r* = .46; .13). It should be noted that the item total correlations in the normative sample (SD [Fisher-Z(*r*)] = .08) vary considerably less than in the clinical sample (SD[Fisher-Z(*r*)] = .13), so that the strength of the correlation is impaired. Generally, for those items with the highest information redundancy, the estimation with EM is carried out with the lowest error in both data sets. Since the assumption of the MD process MAR is most plausible for the items with the highest information redundancy, it substantiates the underlying assumptions of MD theory (Allison, 2001; Enders, 2010; Graham, 2009). The results determined here thus also provide information on the suitability of EM imputation for other multi-item scales: The higher the internal consistency of the scales and the higher the discriminatory power of the scale items, the more valid and precise the imputation should be, since the assumption of MAR, that the probability of missing responses can be modelled by the other scale items, is more plausible. These findings support the general assumption, that the problem of missing items is in general less critical with multi-item questionnaires which exhibit a high internal consistency, due to the systematic dependence of the items on each other and on the latent trait, respectively (Peyre et al., 2011; Shrive et al., 2006). Generally, it might be an interesting challenge for future research to systematically study the relationship between the psychometric properties of scales (in particular internal consistency or correspondence to standards of classical test theory or item response theory), and the appropriateness of the MD-process assumption MAR or imputation quality, respectively.

As potential limitation of this study, it should be considered that we focused on imputation of single missing item responses, because MD diagnostics revealed no salient MD patterns. Because the items of SF-12 were explicitly selected to represent the multidimensional structure of SF-36 as distinctly as possible by single items or item pairs, other studies have shown that patterns of MD are of secondary importance (Liu et al., 2005; Morfeld et al., 2003; Perneger & Burnand, 2005). Future research should address the question of whether the superiority of the EM imputation in terms of estimation accuracy on single item level can be confirmed in the case of typical patterns of missing values. As in most multivariate analysis techniques established for the SF-12 (Gandek et al., 1998; Ware et al., 1996, Ware et al., 2001), the EM algorithm assumes normal distributed data (Graham, 2009). However, the EM algorithm proved to be considerably robust against violations (Koch, 2013). Furthermore, no test exists to decide whether the MD process is only MAR and not nonrandom missing. Because the SF-12 items are part of internal consistent scales, the items are highly mutually interdependent. Hence, MAR can be assumed to be at least approximately sufficiently valid.

## Conclusions

The occurrence of MD in the SF-12 is associated with participants’ age and educational background, but not with their physical and mental health state. As MD do not occur completely at random (MCAR), the previously recommended procedures case-wise deletion or mean imputation cause systematic biases. The EM algorithm provides the most valid technique to handle MD in the SF-12, as it allows for the highest concordance of imputed and real data in a clinical and a normative study sample on single item level. Using the EM algorithm can be recommended as mandatory in case of MD in the SF-12. These findings are not only valuable to enhance recommendations for the optimal handling of missing values in the SF-12. Rather, the SF-12 is an example of multi-item scales that measure patient-reported HRQoL. The evaluation method and the findings may thus be representative of a high-quality procedure for empirically analyzing and justifying an adequate handling of the problem of missing values in HRQoL assessments in clinical and epidemiological studies.
